# Effects of unfolding case-based simulation in obstetrics and gynecology internships on caring ability and caring behaviors: a quasi-experimental study

**DOI:** 10.3389/fmed.2026.1815304

**Published:** 2026-06-12

**Authors:** Meiqi Yao, Qi Wu, Zhuqing Wang, Longfang Ni, Fang Liao, Xiaohong Zhu, Sibei Wan, Wenjie Wang, Lina Yang

**Affiliations:** 1 Department of Nursing, Shanghai Sixth People’s Hospital Affiliated to Shanghai Jiao Tong University School of Medicine, Shanghai, China; 2 School of Medicine, Tongji University, Shanghai, China; 3 Shanghai General Hospital, Shanghai, China

**Keywords:** caring ability, caring behaviors, clinical internship, nursing students, obstetrics and gynecology nursing, quasi-experimental study, unfolding case–based simulation

## Abstract

**Background:**

Obstetrics and gynecology (OBGYN) internships are emotionally demanding and require nursing students to integrate specialty competence with sustained caring. Evidence on whether unfolding case–based simulation can improve both caring ability and caring behaviors during OBGYN internships remains limited.

**Methods:**

We conducted a prospective, controlled quasi-experimental study in the OBGYN ward of Shanghai Sixth People’s Hospital from June 2024 to March 2025. Final-year undergraduate nursing interns (*n* = 106) completed a 4-week rotation and were allocated by individual-level lot drawing within naturally occurring rotation cohorts to a control group receiving conventional preceptorship (*n* = 47) or an intervention group receiving conventional preceptorship plus unfolding case–based simulation (*n* = 59). Primary outcomes were end-of-rotation Caring Ability Inventory (CAI) and Caring Behaviors Inventory (CBI) scores, analyzed using analysis of covariance with group as the fixed factor and the corresponding baseline score as a covariate. Secondary outcomes included end-of-rotation specialty assessments and teaching satisfaction.

**Results:**

Baseline characteristics and baseline CAI and CBI scores were comparable between groups. After adjustment for baseline scores, the intervention group had significantly higher end-of-rotation CAI and CBI total and dimension scores than the control group (all *P* < 0.001). The intervention group also achieved higher specialty assessment scores and overall teaching satisfaction than the control group (both *P* < 0.001).

**Conclusion:**

Adding unfolding case–based simulation to conventional OBGYN preceptorship was associated with substantial short-term improvements in nursing students’ caring ability and caring behaviors, alongside better specialty performance and teaching satisfaction. Further studies using multicenter designs, delayed follow-up, and objective behavioral assessments are needed to determine whether these gains are sustained and transferable to real clinical practice.

## 1 Introduction

As medical education reform advances and clinical care becomes increasingly complex, nursing education has shifted from a primary focus on technical skill acquisition to competency development (1). Clinical internship is a pivotal stage during which nursing students integrate knowledge, skills, and professional attitudes, thereby shaping their clinical competence, professional identity, and implementation of patient-centered care (2). These challenges are particularly evident in obstetrics and gynecology (OBGYN), where rapid changes in patients’ conditions, high-risk clinical situations, frequent ethical decisions, and substantial emotional burdens for patients and families require students to not only master specialty-specific skills but also demonstrate stable communication, empathy, and caring behaviors under pressure (2).

At present, clinical teaching in OBGYN remains largely task-oriented, typically relying on bedside instruction, procedural demonstration, and one-to-one clinical shadowing. Although this approach supports the development of fundamental technical skills, it has structural limitations in fostering students’ integration of higher-order competencies, including clinical reasoning, communication and negotiation, ethical judgment, and humanistic care. Traditional preceptorship often emphasizes how to perform tasks but provides less systematic training in why certain actions are taken, how to engage patients and families in shared decision-making, and how to sustain caring behaviors in the face of clinical conflicts and stress. Caring in nursing is not merely an affective attitude but a relational and behavioral process enacted through understanding, emotional presence, supportive action, and empowerment (3, 4). Watson’s Theory of Human Caring provides a philosophical foundation for viewing caring as an intentional, human-to-human process that should be cultivated in nursing education (4). Swanson’s Theory of Caring further operationalizes caring into five processes—maintaining belief, knowing, being with, doing for, and enabling—which are particularly relevant to clinical teaching because they translate caring into observable interactions with patients and families (5).

In this study, caring ability and caring behaviors were treated as related but distinct constructs. Caring ability refers to a learner’s internal capacity and disposition to establish caring relationships, understand patients’ needs, respond to uncertainty, and sustain patient-centered attitudes. This construct was assessed using the Caring Ability Inventory (CAI), developed by Nkongho, which measures three dimensions: cognition, courage, and patience (6). In contrast, caring behaviors refer to the observable enactment of caring in clinical encounters, including respectful interaction, support and assurance, and the use of professional knowledge and skills to meet patients’ needs. This construct was assessed using the Caring Behaviors Inventory (CBI), developed by Wolf, which focuses on the behavioral expression of caring in nursing practice (7). Therefore, improvement in CAI represents development in students’ internal caring capacity, whereas improvement in CBI represents improvement in the external performance of caring behaviors.

To translate these constructs into teachable and assessable actions, we developed an intervention-specific CARE framework for the unfolding case–based simulation: Connect, Acknowledge, Restructure, and Empower. Each component was designed to target both caring ability and caring behaviors, but at different levels. “Connect” aimed to strengthen students’ capacity for emotional presence and perspective-taking while training observable behaviors such as initiating trustful communication and responding respectfully to patients and families. “Acknowledge” targeted students’ ability to recognize patients’ emotional and decisional concerns, while requiring them to enact empathic listening, validation, and supportive explanation. “Restructure” was intended to develop students’ courage and judgment in managing family conflict and uncertainty, while training behaviors such as reframing family roles, clarifying care goals, and supporting shared decision-making. “Empower” aimed to enhance students’ ability to promote patients’ perceived control, while training behaviors such as providing understandable information, using visual aids, and encouraging patient participation in care decisions. In this way, the CARE framework served as the operational bridge between caring theory, the CAI construct of caring ability, and the CBI construct of caring behaviors.

It should be clearly pointed out that the CARE framework constructed in this study is not a direct operational mapping of Watson and Swanson’s caring theory, nor does it assume a strict one-to-one correspondence between its constituent elements (Connect, Acknowledge, Restructure, Empower) and each dimension of the CAI or CBI scales. Designed to serve dynamic case simulation teaching, the CARE framework acts as a specific and practical action guideline, with the core goal of converting abstract caring concepts into teachable and assessable clinical tasks. Accordingly, this framework represents a pedagogical re-creation grounded in relevant caring theories. It aims to comprehensively improve learners’ caring competence and behaviors through integrated practice in simulated scenarios, rather than precisely modifying a single specific dimension in the scales via individual framework elements.

Teaching strategies grounded in constructivism and situated cognition—such as simulation-based learning, problem-based learning (PBL), and scenario-based instruction—have been shown to improve learner engagement, self-directed learning, and clinical reasoning (8). However, when the educational goal extends from technical competence to caring ability and caring behaviors, instructional design must provide learners with opportunities to enact caring responses in authentic interpersonal and ethical situations. Much of the existing simulation instruction remains based on static, single-stage, or task-completion scenarios, which may not adequately capture the dynamic trajectories of clinical practice, including evolving family interactions and escalating ethical conflicts (9). This limitation is particularly salient in OBGYN nursing, where a single case may rapidly unfold into a sequence of events such as clinical deterioration, disagreements among family members, time-sensitive intrapartum decisions, and uncertainty regarding neonatal outcomes (10). Without exposure to such dynamic stressors and structured reflection, students may understand caring as an abstract professional value but struggle to convert it into stable, context-sensitive caring behaviors in practice (11).

Building on this framework, situated cognition and experiential learning informed the simulation design by embedding CARE-aligned caring actions into authentic, evolving OBGYN scenarios. Unfolding case–based simulation is an experiential learning approach that uses multi-episode, progressively unfolding case scripts to embed clinical deterioration, communication conflict, and ethical dilemmas into a continuous context (12). In the present study, this approach was used as the pedagogical vehicle through which the CARE framework could be practiced in dynamic OBGYN scenarios. Conceptually, unfolding case–based simulation may promote caring ability by exposing students to emotionally charged and uncertain situations that require understanding, courage, and patience. At the same time, it may promote caring behaviors by requiring students to repeatedly perform concrete caring actions, including respectful communication, emotional support, family negotiation, patient education, and empowerment. Structured debriefing further supports this process by helping students reflect on the gap between caring intention and caring behavior, thereby converting simulated experience into transferable behavioral scripts (13). Nevertheless, evidence regarding the effectiveness of unfolding case–based simulation during OBGYN clinical internships remains limited. In particular, controlled studies that simultaneously evaluate caring ability and caring behaviors, while also considering professional competence and learning experiences, are scarce.

Guided by this conceptual distinction between caring ability and caring behaviors, we conducted a prospective, controlled quasi-experimental study during OBGYN clinical internships to compare conventional preceptorship alone with conventional preceptorship plus unfolding case–based simulation. We evaluated nursing students’ caring ability using the Caring Ability Inventory (CAI), caring behaviors using the Caring Behaviors Inventory (CBI), OBGYN specialty assessments, and teaching satisfaction. We hypothesized that, compared with the control group, students receiving unfolding case–based simulation would demonstrate higher end-of-rotation CAI and CBI scores after adjustment for baseline scores, as well as better specialty assessment performance and higher teaching satisfaction. This study aimed to provide a replicable and evaluable instructional pathway for clinical education in emotionally demanding specialties and to offer empirical support for the coordinated development of caring ability, caring behaviors, and specialty-specific competence.

## 2 Materials and methods

### 2.1 Study design

This prospective, controlled, quasi-experimental study evaluated whether unfolding case–based simulation could improve nursing students’ caring ability and caring behaviors during an OBGYN clinical internship. Outcomes were assessed at two time points: at the start of the rotation (baseline) and at the end of the 4-week rotation. The primary outcomes were end-of-rotation CAI and CBI scores, analyzed with adjustment for the corresponding baseline scores. Pre–post changes were reported descriptively to show within-student score changes over the 4-week rotation. Secondary outcomes included end-of-rotation OBGYN specialty assessment scores (theory and skills) and teaching satisfaction. This study is reported in accordance with the TREND (Transparent Reporting of Evaluations with Nonrandomized Designs) statement.

### 2.2 Study setting and participants

This study was conducted in the OBGYN ward of Shanghai Sixth People’s Hospital, Shanghai Jiao Tong University School of Medicine, Shanghai, China, from June 2024 to March 2025. Participants were recruited at the beginning of each rotation cohort and followed throughout the 4-week internship. Convenience sampling was used based on cohort size and teaching feasibility. No *a priori* sample size calculation was performed. However, a *post hoc* power analysis indicated that with a total sample size of 106 participants (47 vs. 59), the study had approximately 80% power to detect a moderate between-group effect size (Cohen’s *d* ≈ 0.55) at a two-sided α level of 0.05. In total, 106 final-year undergraduate nursing students undertaking OBGYN clinical internships were enrolled. All participants completed the rotation and study assessments; no missing data were observed.

Inclusion criteria were: (1) final-year nursing interns from universities in Shanghai; (2) completion of foundational medical and nursing coursework; (3) no prior exposure to unfolding case–based simulation; and (4) provision of informed consent.

Exclusion criteria were: (1) failure to complete the OBGYN internship program or key assessments due to sick leave or personal leave; and (2) prior learning experiences highly similar to unfolding case–based simulation.

Grouping method: Interns entered the OBGYN rotation in naturally occurring cohorts according to routine teaching schedules. Within each cohort, allocation was performed at the individual level using a lot-drawing procedure conducted by the teaching coordinator. The teaching coordinator was not involved in outcome assessment. Group A received conventional clinical preceptorship and served as the control group, whereas Group B received conventional preceptorship plus unfolding case–based simulation and served as the intervention group. The resulting group sizes were 47 in the control group and 59 in the intervention group. Because students remained embedded in routine rotation cohorts and allocation concealment was not equivalent to that of a fully randomized controlled trial, the study was reported as a controlled quasi-experimental study.

**FIGURE 1:**
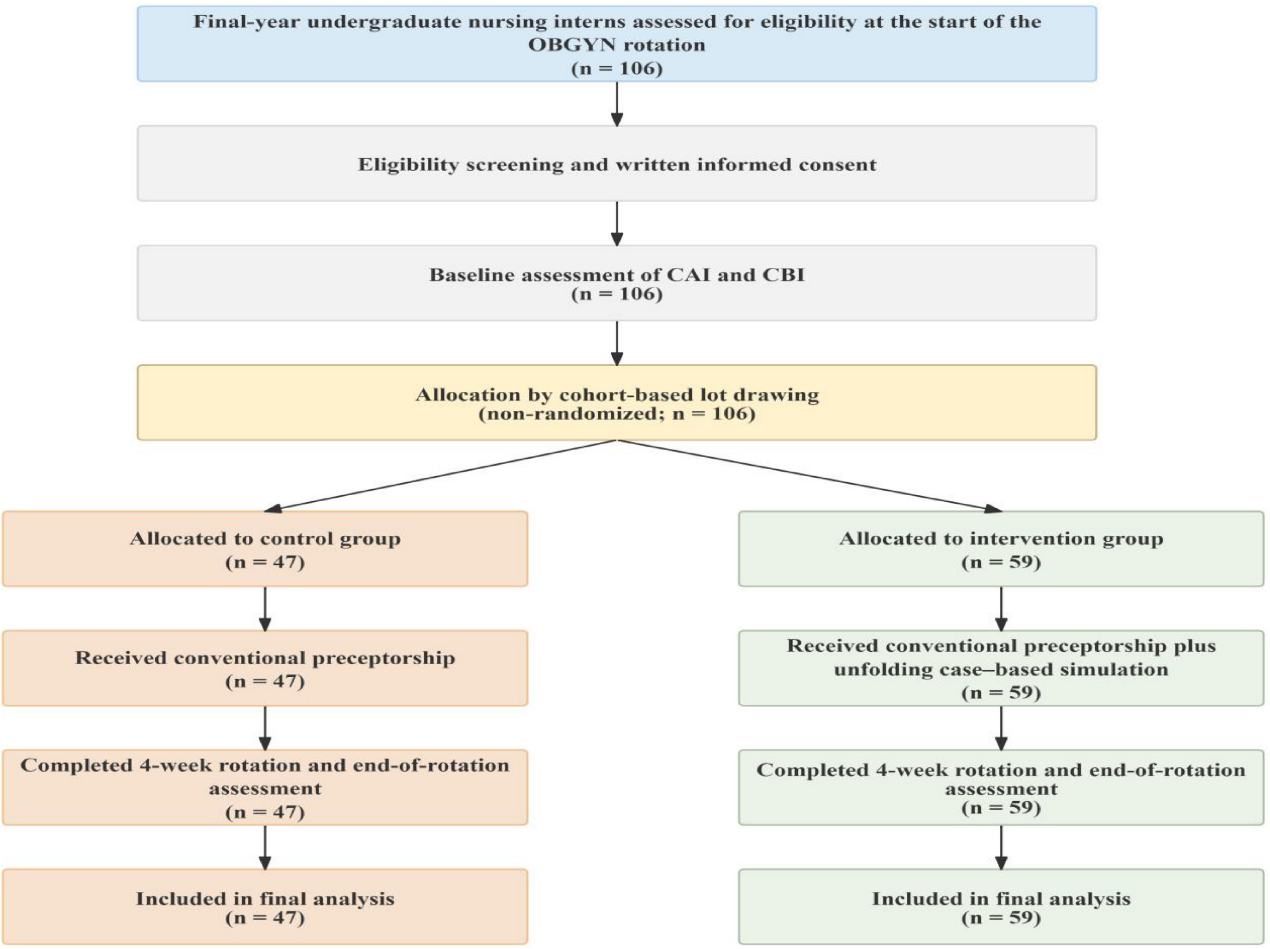
Participant flow diagram. OBGYN, obstetrics and gynecology; CAI, Caring Ability Inventory; CBI, Caring Behaviors Inventory.

The participant flow is shown in Figure 1.

### 2.3 Educational content and intervention

#### 2.3.1 Control group

The control group received a conventional preceptorship model led by clinical preceptors and focused on knowledge and skills acquisition. A unified teaching plan was developed by the ward in accordance with the school’s internship syllabus and delivered via one-to-one, shift-based preceptorship. Bedside instruction and procedural demonstration were the main teaching formats. A weekly mini-lecture was provided, and a case discussion was organized monthly. Teaching content covered OBGYN specialty theory, procedural skills, key observation points, and routine communication skills.

#### 2.3.2 Intervention group

In addition to conventional preceptorship, the intervention group received unfolding case–based simulation guided by the theoretical framework described in the Introduction. This experiential approach, informed by situated cognition and structured around the intervention-specific CARE framework, used progressively unfolding, multi-episode cases to guide learners’ assessment, communication, decision-making, caring responses, and reflection in simulated OBGYN scenarios.

##### 2.3.2.1 Teaching team establishment and training

A dedicated teaching team was established, consisting of two charge nurses, one teaching coordinator, and four senior midwives or registered nurses. All members had more than 10 years of OBGYN clinical experience and more than 5 years of teaching experience. Before implementation, all members received standardized training in unfolding case design and facilitation, with an emphasis on consistent scenario delivery and structured debriefing.

##### 2.3.2.2 Case development and resource preparation

The teaching team developed one standardized unfolding case script based on the textbook Obstetrics and Gynecology Nursing (People’s Medical Publishing House) and common high-risk conditions encountered in the OBGYN ward. Hypertensive disorders of pregnancy were selected as the core scenario because they involve dynamic maternal–fetal risk assessment, treatment decision-making, family communication, emotional support, and ethical tension, making them suitable for training both clinical reasoning and caring responses. During case development, each scenario phase was mapped to one or more CARE components to ensure that clinical tasks, communication challenges, family interactions, and reflective prompts were explicitly linked to caring ability and caring behavior training. The standardized script covered a sequence of dynamic scenarios from admission and clinical deterioration to delivery decision-making and postpartum care, and incorporated common psychosocial conflicts and ethical dilemmas (Table 1). Supporting micro-lecture videos were recorded, learning manuals were prepared, and all materials were uploaded to the online teaching platform.

**TABLE 1:** Standardized unfolding case–based simulation process: hypertensive disorders of pregnancy.

Phase	Nursing tasks	Standardized patient and family responses (SP, standardized patient)
Case background	Ms. Yu, 28 years old, G2P0, 34+2 weeks of gestation, conceived by IVF, with gestational hypertension. Occupation: University teacher (well-educated, high information needs). Personality: Anxious and sensitive, with repeated inquiries about fetal safety. Caregiver: Mother-in-law (husband away for work and unable to arrive promptly).	Detail: Her mobile phone screensaver is a three-dimensional color Doppler ultrasound image of the fetus, suggesting strong emotional investment.
Phase	Nursing tasks	Standardized patient and family responses (SP, standardized patient)
Phase 1: admission assessment	Measure vital signs on emergency admission to the ward (blood pressure 150 mmHg systolic and 100 mmHg diastolic; mild edema of the lower extremities).	The patient repeatedly asks, “Nurse, my blood pressure is so high. Will my baby be okay?”
	Venous blood sampling; assist with electrocardiographic monitoring and fetal monitoring.	The mother-in-law repeatedly interrupts nursing procedures, saying, “Where is the doctor? You must ensure my grandchild is safe.”
Phase 2: magnesium sulfate therapy	Establish peripheral intravenous access (indwelling catheter).	The patient asks, “After taking this medicine, will my blood pressure improve?”
	Prepare and administer magnesium sulfate; monitor patellar reflex, respiratory rate, and urine output.	The mother-in-law asks, “Will this medicine harm the baby?”
	Use the infusion pump.	The patient reports, “I feel hot, and my chest feels tight. I cannot catch my breath.”
Conflict	Physician orders: blood pressure rises to 160 mmHg systolic and 110 mmHg diastolic; proteinuria (++); suspected preeclampsia; fetal monitoring shows recurrent late decelerations. Prepare for possible termination of pregnancy and continuous maternal and fetal monitoring.	The husband is unable to arrive at the hospital promptly.
Phase 3: delivery decision-making	Eclampsia prophylaxis	The mother-in-law refuses to sign the consent form, saying, “The baby is not full term. Wait for my son to come.”
	Preoperative preparation (surgical skin preparation, blood product readiness, instrument and supply readiness)	The patient becomes emotionally overwhelmed, saying, “I conceived through several attempts. The baby must be safe…”
Conflict	Preterm newborn apgar scores and management The preterm newborn exhibited Apgar scores of 7 at 1 min (components: respiration, muscle tone, and color each deducted 1 point) and 8 at 5 min (components: respiration and color each deducted 1 point). Due to persistent clinical concerns, the infant was transferred to the Neonatal Intensive Care Unit (NICU) for further observation and management.	The mother-in-law cooperates with the admission procedures.
Phase 4: postpartum monitoring	Creation and donation of newborn footprint cards to the delivery room	The parturient asked, “I have not yet seen my baby. What does he look like?”
	Establishment of video connection with the NICU.	The parturient asked, “How is my baby doing in the NICU?”

##### 2.3.2.3 Teaching implementation process

(1) Preparation phase (week 1): At the start of the rotation, pre-recorded videos and case materials were uploaded to the online teaching platform. Interns were instructed to complete the video-based learning prior to in-person sessions. An online discussion group was established to facilitate communication and to collect questions raised during self-study.
(2) Theoretical consolidation phase (week 2): Based on common issues identified during preparation, preceptors delivered an in-person session with interactive discussion to consolidate disease-related knowledge, key nursing points, and principles of humanistic communication. Based on the theoretical framework described in the Introduction, preceptors introduced the intervention-specific CARE framework as an operational guide for humanistic caring practice. The four components—Connect, Acknowledge, Restructure, and Empower—were explained using OBGYN examples, including establishing emotional connection, validating patients’ concerns, supporting decision-making, reframing family roles during conflict, and enhancing patients’ perceived control through clear explanations, visual aids, and supportive communication.
(3) Simulation practice and debriefing phase (week 3): A half-day unfolding case–based simulation was conducted in a simulated delivery room or ward (late in Week 3). The same standardized case script was used for all intervention participants to ensure consistency of intervention delivery. The case unfolded dynamically and emphasized integrated clinical thinking that combines knowledge, skills, and communication competence. Preceptors role-played standardized patients, family members, and physicians. In each round, three interns assumed roles (e.g., primary nurse and midwife), while other interns observed. Interns received a script containing partial information and were required to conduct real-time assessment, communication, decision-making, and nursing implementation in response to changes in patient status, family reactions, and physicians’ orders. Immediately after the simulation, a structured debriefing was conducted: participating interns completed self-review and reflection; observers provided feedback; and the teaching coordinator facilitated a group discussion focused on clinical judgment, communication effectiveness, humanistic caring practice, and ethical decision-making, followed by a summary of key learning points.
(4) Assessment and summary phase (Week 4): Interns’ integrated performance in knowledge, skills, and humanistic caring competence was evaluated through summative assessments and structured reflection. Early in Week 4, interns completed a closed-book OBGYN theoretical examination (maximum score 100) and a skills assessment. The skills assessment was rated using a standardized rubric by two senior nurses who had received unified training and were blinded to group allocation. After the assessments, a structured reflection seminar was held. Each group shared learning gains and reflections on typical cases, followed by preceptors’ feedback and synthesis. Finally, 1–2 days before completion of the rotation, interns completed the questionnaires using self-generated identification codes, which allowed linkage of baseline and end-of-rotation responses while ensuring that no personally identifiable information was collected.

### 2.4 Evaluation of teaching outcomes

#### 2.4.1 OBGYN specialty assessment scores

The OBGYN theoretical examination consisted of objective items (single-choice, multiple-choice, and true-or-false questions) with a maximum score of 100. Test content was derived from the textbook Obstetrics and Gynecology Nursing and the OBGYN section of the national nursing licensure examination question bank. The skills assessment comprised one basic skill and one specialty-specific skill. The total skills score (maximum 100) was calculated as 50% for the basic skill and 50% for the specialty-specific skill. The overall specialty assessment score (maximum 100) was calculated as 50% theory and 50% skills.

#### 2.4.2 Teaching satisfaction questionnaire for nursing interns

Teaching satisfaction was assessed using the institution’s standardized nursing intern teaching satisfaction questionnaire. The questionnaire comprised 10 items: (1) preceptors’ professional role modeling and ethical conduct; (2) attention to the implementation and scheduling of the internship teaching plan; (3) practicality of teaching content and integration of theory with practice; (4) development of interns’ practical working ability; (5) fostering of creative thinking; (6) enhancement of comprehensive ability; (7) provision of humanistic caring and psychological support; (8) individualized teaching tailored to learners’ differences; (9) provision of learning opportunities and a supportive learning environment; and (10) mechanisms for teaching feedback and continuous improvement. Each item was rated from 0 (extremely dissatisfied) to 10 (extremely satisfied). Total scores ranged from 0 to 100, with higher scores indicating greater satisfaction.

#### 2.4.3 Caring Ability Inventory (CAI)

Caring ability was assessed using the CAI developed by Nkongho and translated and culturally adapted into Chinese by Ma Fang (14). The scale includes three dimensions (Cognition, Courage, and Patience) and 37 items. Items are rated on a seven-point Likert scale from 1 (strongly disagree) to 7 (strongly agree), yielding a total score range of 37–259. Higher scores indicate stronger humanistic caring ability. In the present sample, Cronbach’s alpha was 0.832 for the total scale and 0.785, 0.720, and 0.748 for the three dimensions, indicating good internal consistency. In this study, CAI was used to capture the internal dimension of caring development, corresponding to students’ understanding of caring, willingness to engage in caring relationships, and capacity to maintain patient-centered responses in challenging clinical situations (15).

#### 2.4.4 Caring Behaviors Inventory (CBI)

Caring behaviors were assessed using the CBI developed by Wolf (7) and translated into Chinese by Da Chaojin (14). The CBI comprises 24 items rated on a six-point Likert scale from 1 (never) to 6 (frequently), yielding a total score range of 24–144; higher scores indicate better caring behaviors. In this study, we reported the total CBI score and three domain scores (Support and assurance, Knowledge and skills, and Respect for patients). Cronbach’s alpha was 0.959 for the total scale, with subscale alphas ranging from 0.897 to 0.928. In this study, CBI was used to capture the behavioral expression of caring, corresponding to students’ observable respect, support, assurance, knowledge-based care, and patient-centered interaction during clinical encounters (16).

### 2.5 Data collection and quality control

Both groups completed the CAI and CBI at baseline (start of rotation) and at the end of the 4-week rotation. At rotation end, additional outcomes were collected, including OBGYN specialty assessment scores (theory and skills) and teaching satisfaction. Data were entered with double-checking and logic checks; a random subset of records was audited, and discrepancies were resolved by a third reviewer when necessary. To minimize contamination between groups, core teaching activities were scheduled at different times and conducted in separate spaces, and the two groups were guided by different preceptors. During orientation, interns were instructed not to discuss specific teaching components across groups. Intervention fidelity and separation were monitored through routine supervision by the teaching coordinator. To enable within-person analysis, participants generated unique, self-identifying codes that were used to match baseline and post-rotation responses; the dataset was de-identified prior to analysis. Baseline CAI and CBI assessments were completed prior to group allocation, and participants were not informed of their group assignment at the time of baseline measurement.

### 2.6 Bias control

To reduce selection bias, standardized inclusion and exclusion criteria were applied, and baseline characteristics were compared between groups to confirm comparability (see Table 2). To control information bias, CAI, CBI, specialty assessments, and the teaching satisfaction questionnaire were administered by trained investigators following standardized procedures at fixed time points. Theoretical examinations used unified test papers, and the skills assessment was rated by senior nurses who were blinded to group allocation and had received standardized training. Data entry used double-checking and logic verification. To reduce analysis bias, statistical analyses were performed by an analyst blinded to group allocation.

**TABLE 2:** Baseline characteristics of nursing interns by group (*n* = 106).

Item	Intervention group (*n* = 59)	Control group (*n* = 47)	*t/χ* ^2^	*P*
Gender (*n*, %)			0.769	0.381
Male	19 (32.20)	19 (40.40)	–	–
Female	40 (67.80)	28 (59.60)	–	–
Age (years)	21.78 ± 0.83	21.61 ± 0.92	0.998	0.320
Educational level (*n*, %)			1.055	0.304
Junior college or below	23 (39.00)	23 (48.90)	–	–
Bachelor degree or above	36 (61.00)	24 (51.10)	–	–
Only child (*n*, %)			0.209	0.647
Yes	52 (88.10)	40 (85.10)	–	–
No	7 (11.90)	7 (14.90)	–	–
Political status (*n*, %)			0.673	0.714
League member	42 (71.20)	36 (76.60)	–	–
Party member (probationary member)	11 (18.60)	6 (12.80)	–	–
Others	6 (10.20)	5 (10.60)	–	–
Volunteer service participation (*n*, %)			1.364	0.243
Yes	31 (52.50)	30 (63.80)	–	–
No	28 (47.50)	17 (36.20)	–	–

### 2.7 Statistical methods

Data were analyzed using IBM SPSS Statistics version 27.0. Normally distributed continuous variables are presented as mean ± standard deviation and were compared using independent-samples *t*-tests. Non-normally distributed continuous variables are presented as median (Q1, Q3) and were compared using the Mann-Whitney U test. Categorical variables are presented as *n* (%) and were compared using the chi-square test. For CAI and CBI outcomes, post-intervention scores were compared between groups using analysis of covariance (ANCOVA), with group as the fixed factor and the corresponding baseline score as a covariate. The homogeneity of regression slopes assumption was examined by testing the group × baseline interaction. A two-sided *P*-value < 0.05 was considered statistically significant. Item-level analyses of teaching satisfaction were considered exploratory and hypothesis-generating. To account for multiple comparisons across the 10 satisfaction items, Benjamini-Hochberg false discovery rate correction was performed as a sensitivity analysis.

### 2.8 Ethical considerations

This study was approved by the Institutional Review Board of Shanghai Sixth People’s Hospital, Shanghai Jiao Tong University School of Medicine (Approval No. X20240114) prior to data collection. All procedures complied with institutional ethics requirements and the Declaration of Helsinki. Written informed consent was obtained from all interns after they received a full explanation of the study. Study data were de-identified prior to analysis, used solely for research purposes, and stored securely in accordance with institutional data management policies. This study was not prospectively registered. As a quasi-experimental educational study without randomization or clinical intervention, formal trial registration was not performed. To minimize potential coercion due to the student–preceptor relationship, questionnaire completion was voluntary and conducted without the presence of teaching staff.

## 3 Results

### 3.1 Baseline characteristics

In total, 106 nursing interns were included (intervention: *n* = 59; control: *n* = 47). The two groups were comparable at baseline in terms of demographic and training characteristics, including sex, age, educational level, only-child status, political status, and volunteer service participation (all *P* > 0.05; Table 2).

### 3.2 End-of-rotation specialty assessment and teaching satisfaction

At the end of the rotation, the intervention group outperformed the control group on the specialty assessment, including total scores and all subdomains (all *P* < 0.001). Regarding teaching satisfaction, the intervention group also scored higher on most items, with no significant differences observed for “professional role modeling and ethical conduct of preceptors” (*P* = 0.186) or “attention to the implementation of the internship teaching plan” (*P* = 0.119) (Table 3). Because the 10 individual satisfaction items were considered exploratory and hypothesis-generating, a sensitivity analysis using the Benjamini–Hochberg false discovery rate correction was performed. After correction, items 3–10 remained statistically significant, whereas items 1 and 2 remained non-significant, indicating that the item-level findings were generally robust but should still be interpreted as exploratory.

**TABLE 3:** End-of-rotation specialty assessment and teaching satisfaction scores by group.

Item	Intervention group (*n* = 59)	Control group (*n* = 47)	*Z*	*P*
Specialty assessment	93.75 (92.50, 95.25)	91 (89.75, 91.50)	−8.334	<0.001
Theoretical knowledge	94 (93, 96)	92 (90, 92)	−6.752	<0.001
Operational skills	93.5 (92, 95)	90.5 (89.50, 91)	−8.433	<0.001
Specialized operations	93 (92, 95)	90 (89, 92)	−7.322	<0.001
Basic operations	94 (93, 95)	91 (90, 91)	−7.326	<0.001
Total score of teaching satisfaction questionnaire	98 (97, 99)	94 (93, 95)	−7.834	<0.001
1. Professional role modeling and ethical conduct of preceptors	10 (10, 10)	10 (9, 10)	−1.323	0.186
2. Attention to the implementation of internship teaching plan	10 (10, 10)	10 (9, 10)	−1.557	0.119
3. Practicality of teaching content and integration of theory with practice	10 (10, 10)	10 (9, 10)	−3.425	0.001
4. Development of students’ practical working ability	10 (9, 10)	9 (8, 9)	−6.045	<0.001
5. Cultivation of students’ creative thinking	10 (9, 10)	9 (8, 9)	−5.405	<0.001
6. Cultivation of students’ comprehensive ability	10 (9, 10)	10 (8, 10)	−3.172	0.002
7. Humanistic care and psychological support for students	10 (10, 10)	9 (9, 10)	−4.246	<0.001
8. Individualized and differentiated teaching according to students’ aptitude	10 (9, 10)	9 (9, 10)	−2.991	0.003
9. Provision of learning opportunities and creation of an engaging learning environment	10 (9, 10)	9 (9, 10)	−4.842	<0.001
10. Teaching feedback and continuous improvement mechanism	10 (9, 10)	9 (8, 10)	−4.791	<0.001

Data are presented as median (Q1, Q3). Between-group comparisons were performed using the Mann-Whitney U test. Z denotes the standardized test statistic. *P*-values are two-sided. The 10 individual satisfaction items were exploratory and hypothesis-generating. Benjamini-Hochberg false discovery rate correction was applied as a sensitivity analysis; items 3–10 remained statistically significant after correction, whereas items 1 and 2 remained non-significant.

### 3.3 Baseline CAI and CBI scores at the start of the OBGYN rotation

At baseline, CAI and CBI total and dimension scores were comparable between groups (all *P* > 0.05; Table 4).

**TABLE 4:** Baseline Caring Ability Inventory (CAI) and Caring Behaviors Inventory (CBI) total and dimension scores by group (*n* = 106).

Item	Intervention group (*n* = 59)	Control group (*n* = 47)	*t*	*P*	Hedges’ *g*	95% CI
Total CAI score	185.43 ± 14.80	184.39 ± 12.45	0.391	0.696	0.075	(−0.306, 0.455)
Cognition	74.87 ± 5.48	73.69 ± 6.84	0.960	0.339	0.191	(−0.190, 0.573)
Courage	55.11 ± 11.29	55.19 ± 8.62	0.041	0.967	−0.008	(−0.388, 0.373)
Patience	55.45 ± 5.41	55.51 ± 6.40	0.053	0.958	−0.010	(−0.391, 0.370)
Total CBI score	108.35 ± 7.24	106.61 ± 7.56	1.204	0.231	0.234	(−0.148, 0.616)
Respect for patients	46.46 ± 4.20	45.21 ± 3.73	1.594	0.114	0.310	(−0.072, 0.693)
Knowledge and skills	23.49 ± 2.71	22.63 ± 2.90	1.541	0.126	0.305	(−0.077, 0.688)
Support and assurance	38.40 ± 4.32	38.76 ± 4.31	0.428	0.669	−0.083	(−0.463, 0.298)

Data are presented as mean ± SD. Between-group comparisons were performed using independent-samples *t*-tests. Hedges’ *g* was calculated as the standardized mean difference (Intervention–Control) with small-sample correction; 95% CIs are shown. *P-*values are two-sided.

### 3.4 Baseline-adjusted between-group differences in CAI and CBI outcomes

Raw pre-post change scores showed larger improvements in the intervention group than in the control group for CAI and CBI total and dimension scores. In the primary baseline-adjusted analysis using ANCOVA, the intervention group had significantly higher end-of-rotation CAI and CBI total and dimension scores than the control group after adjustment for the corresponding baseline scores (all *P* < 0.001; Table 5). The adjusted mean differences favored the intervention group for total CAI score, total CBI score, and all subdomains, and all 95% confidence intervals excluded zero. Partial η^2^ values indicated large effects for total CAI, cognition, courage, total CBI, and respect for patients, and smaller but still significant effects for patience, knowledge and skills, and support and assurance.

**TABLE 5:** Raw pre-post change scores and baseline-adjusted between-group differences in Caring Ability Inventory (CAI) and Caring Behaviors Inventory (CBI) outcomes.

Item	Intervention group change score (*n* = 59)	Control group change score (*n* = 47)	Baseline-adjusted mean difference	*F*	*P*	95% CI	Partial η^2^
Total CAI score	38.32 ± 9.54	22.45 ± 5.59	15.640	118.231	<0.001	(12.787, 18.493)	0.537
Cognition	11.83 ± 5.65	6.49 ± 3.68	4.809	43.949	<0.001	(3.370, 6.248)	0.301
Courage	17.66 ± 6.91	9.38 ± 3.68	8.299	70.393	<0.001	(6.337, 10.261)	0.408
Patience	8.83 ± 3.77	6.57 ± 2.43	2.255	21.305	<0.001	(1.286, 3.244)	0.173
Total CBI score	21.44 ± 4.74	13.44 ± 3.22	8.256	120.600	<0.001	(6.765, 9.748)	0.542
Respect for patients	9.26 ± 2.21	5.47 ± 2.05	2.231	52.551	<0.001	(1.620, 2.841)	0.340
Knowledge and skills	3.57 ± 1.53	2.70 ± 1.17	1.043	17.263	<0.001	(0.545, 1.541)	0.145
Support and assurance	8.60 ± 3.82	5.26 ± 2.35	2.762	23.691	<0.001	(1.637, 3.888)	0.188

Group-specific values are presented as raw pre-post change scores (mean ± SD) for descriptive purposes. Baseline-adjusted mean differences were obtained using analysis of covariance (ANCOVA), with the end-of-rotation score as the dependent variable, group as the fixed factor, and the corresponding baseline score as a covariate. *F* values, *P*-values, and partial β^2^ correspond to the ANCOVA models. Positive adjusted mean differences indicate higher end-of-rotation scores in the intervention group after baseline adjustment. *P*-values are two-sided.

## 4 Discussion

### 4.1 CARE-guided interpretation of synchronous gains in caring ability and caring behaviors

This study showed that, within a 4-week OBGYN internship, students who received conventional preceptorship supplemented with unfolding case-based simulation demonstrated greater improvements in both CAI and CBI than those receiving conventional preceptorship alone. These findings should be interpreted within the caring-theory framework established in the Introduction. In this framework, caring ability represents students’ internal capacity and disposition to understand patients’ needs, sustain emotional presence, respond to uncertainty, and maintain patient-centered attitudes, whereas caring behaviors represent the observable enactment of caring in clinical encounters. Therefore, the concurrent improvement in CAI and CBI suggests not simply parallel changes in two scale scores, but a potential linkage between students’ perceived internal caring capacity and their self-reported behavioral expression of care. The CARE framework—Connect, Acknowledge, Restructure, and Empower—served as the operational pathway through which this linkage was intentionally trained in the unfolding simulation.

The CBI subscale pattern can be interpreted more coherently through the four CARE components. First, the improvement in Respect for patients may be related to the “Connect” component. In the unfolding case, students were repeatedly required to initiate communication with an anxious pregnant woman and her family, recognize the emotional meaning of fetal safety concerns, and respond before moving directly to task completion. This process may have helped translate the abstract principle of respect into concrete behaviors, including attentive listening, respectful explanation, emotional presence, and recognition of the patient’s subjective experience. From the perspective of caring theory, this can be interpreted as reflecting the movement from knowing about caring to establishing a caring relationship in practice (17).

Second, the improvement in Support and assurance may be closely related to the “Acknowledge” component. The standardized patient and family responses were designed to expose students to repeated worry, interruption, refusal, and uncertainty during maternal–fetal risk escalation, delivery decision-making, and neonatal transfer. These moments required students not only to provide information but also to validate fear, acknowledge decisional distress, and offer reassurance without dismissing patient and family concerns. This suggests that the gains observed in the behavioral domain of support and assurance may be partly attributable to the simulation requiring students to practice empathic responses under pressure rather than merely endorse empathy as a professional value (18).

Third, the “Restructure” component may help explain the relationship between CAI gains, particularly in cognition and courage, and the behavioral expression of care. In the OBGYN context, caring often occurs in situations of conflict among clinical urgency, family expectations, maternal anxiety, and fetal safety concerns (19). The case script required students to reframe the family’s role from passive or oppositional decision-makers to collaborators in urgent care, clarify care priorities, and support shared decision-making (20). Such tasks may potentially strengthen students’ confidence and courage to engage in difficult communication, while also making caring behaviors more structured and clinically actionable. In this sense, CARE can be viewed as a possible bridge between internal caring ability and external caring behavior (21).

Fourth, the “Empower” component may be particularly relevant to the Knowledge and skills subscale of CBI. Although the effect size for Knowledge and skills was smaller than those for Respect for patients and Support and assurance, this domain still improved significantly. This pattern is plausible because knowledge-based caring requires more than emotional support; it requires students to use professional knowledge, visual explanation, risk communication, and procedural competence to help patients understand their condition and participate in care decisions (22). Compared with interpersonal behaviors such as listening or reassurance, this form of caring may require longer clinical exposure and repeated real-case transfer to consolidate. Therefore, the relatively smaller effect in this domain should not be interpreted as definitive evidence of weak intervention impact; rather, it suggests the possibility that professional knowledge and technical competence may require a longer training cycle to be fully integrated into caring behaviors.

The CAI dimension pattern further supports this interpretation. Improvements in cognition and courage may reflect students’ enhanced ability to understand patients’ situations and engage in caring communication despite uncertainty or conflict. In contrast, the relatively smaller effect for patience may indicate that patience is more closely related to emotional regulation, stress tolerance, and sustained interpersonal maturity. These attributes may be less responsive to a single short-cycle intervention and may require longer clinical immersion, repeated feedback, and reflective practice. The dimensional differences across CAI and CBI indicated unfolding case-based simulation yielded large immediate effects on perceived caring cognition, courage, interpersonal respect, and emotional support behaviors, while Patience (CAI) and Knowledge and skills (CBI) showed relatively smaller effect sizes. We propose two testable hypotheses to account for these modest effects for future empirical verification. First, the patience dimension of caring ability is more closely tied to long-term emotional regulation, stress tolerance and professional maturity, which are less susceptible to short-cycle simulation intervention and require prolonged clinical immersion and sustained reflective practice to develop. Second, knowledge-and-skills-oriented caring behavior relies on the integration of professional expertise, risk communication and procedural competency, which cannot be fully consolidated within a single 4-week internship; its improvement requires repeated exposure to real clinical scenarios and longer educational training cycles. These two hypotheses can be explicitly examined in future longitudinal, multi-wave follow-up studies with objective behavioral indicators and extended intervention periods.

Nevertheless, the unusually large effect sizes observed in this study require cautious interpretation. The findings should not be interpreted as definitive evidence that stable caring dispositions or real-world caring behaviors were transformed within a single rotation. Several factors may have amplified the observed effects, including immediate post-intervention assessment, self-reported outcomes, students’ awareness of group assignment, novelty and Hawthorne effects, and close conceptual overlap between the CARE-guided simulation content and the CAI/CBI constructs. Accordingly, these results are best understood as promising short-term improvements in students’ self-reported caring-related outcomes rather than conclusive evidence of durable behavioral transfer to independent clinical practice.

### 4.2 Compatibility between care-oriented simulation and specialty competence development

Our findings also showed that students in the intervention group achieved higher end-of-rotation specialty assessment scores, including theory, skills, and total scores, and reported higher overall teaching satisfaction than students in the control group. These findings suggest that adding CARE-guided unfolding case-based simulation to conventional OBGYN preceptorship was compatible with specialty competence development and perceived learning experience. However, because the present study compared two bundled teaching conditions—conventional preceptorship alone versus conventional preceptorship plus unfolding case-based simulation—it cannot directly determine whether caring-oriented training and specialty competence development had a synergistic relationship (23). Therefore, the findings should be interpreted as co-occurring improvements in caring-related self-reported outcomes, specialty performance, and teaching satisfaction within the intervention condition, rather than as direct evidence of synergy between caring and competence.

One possible explanation for this compatibility is that the unfolding simulation organized learning tasks as a continuous clinical event chain, including disease progression, changing medical orders, family emotions, decision conflicts, nursing interventions, and communication coordination (24). In this format, students were required to extract clinical information, assess maternal–fetal risk, communicate with patients and families, and perform nursing actions within the same scenario. Knowledge and skills were therefore encountered less as fragmented content and more as coherent clinical scripts that were retrievable, applicable, and debrief able. This may help explain why a care-oriented instructional design did not appear to weaken specialty learning and was associated with higher summative assessment performance.

Regarding learning experience, two items—professional role modeling and ethical conduct of preceptors, and attention to the implementation of the internship teaching plan—did not differ significantly between groups, whereas the remaining items and the total teaching satisfaction score favored the intervention group. This pattern suggests that short-cycle educational redesign may be more likely to influence process-related aspects of learning, such as participation opportunities, feedback, perceived support, and competency development, than relatively stable attributes of preceptors or departmental teaching routines (25). Nevertheless, because the item-level satisfaction analyses were exploratory and the satisfaction scores showed potential ceiling effects, these findings should be interpreted cautiously as indicators of perceived learning experience rather than definitive evidence of broad improvement in teaching quality.

Taken together, the specialty assessment and satisfaction findings indicate that CARE-guided unfolding case-based simulation can be integrated into OBGYN internship teaching without an apparent trade-off against specialty learning outcomes (26). Future studies using factorial, dismantling, or mediation designs are needed to determine whether the caring-oriented components, simulation structure, debriefing process, or their interaction independently contribute to improvements in caring-related outcomes and specialty competence.

### 4.3 Implications for curriculum design in emotionally demanding specialties

These findings have practical implications for curriculum design specifically in obstetrics and gynecology and analogous perinatal care contexts. First, caring education should not be positioned as an abstract professional value separated from clinical competence (27). Instead, caring should be embedded into specialty-specific event chains that require students to assess risk, communicate under uncertainty, respond to emotional distress, and perform nursing actions within the same scenario (28). Second, curriculum designers may use a structured framework such as CARE to convert caring theory into observable learning tasks. For example, learning objectives can specify whether students are expected to connect with patients and families, acknowledge emotional and decisional concerns, restructure conflictual expectations, or empower patients through understandable information and participation in care decisions.

Third, emotionally demanding scenarios should include not only clinical deterioration but also relational and ethical turning points, such as family disagreement, refusal of urgent care, fear of adverse outcomes, or distress following transfer to a higher-acuity unit (29). These elements allow students to rehearse caring behaviors at the moment when such behaviors are most likely to deteriorate under pressure. Fourth, debriefing should explicitly evaluate both technical performance and caring performance. Feedback should therefore address not only whether the student selected the correct intervention, but also how the student listened, explained, validated concerns, negotiated family roles, and supported patient participation. Finally, because short-cycle simulation may primarily produce immediate self-reported gains, curriculum designs should consider longitudinal reinforcement rather than relying on a single simulation episode.

### 4.4 Persistence and transfer of caring-related gains beyond the rotation

The 4-week observation window in this study captured immediate post-rotation outcomes; therefore, the persistence of caring ability and caring behaviors beyond the OBGYN rotation cannot be assumed. From the perspective of the CARE framework, short-term simulation may help students form an initial behavioral script for caring practice, including how to connect with patients, acknowledge distress, restructure conflictual expectations, and empower patients through understandable information. However, whether these scripts persist after the rotation depends on continued opportunities for rehearsal, feedback, and reinforcement in subsequent clinical contexts.

Caring-related gains are more likely to be maintained when students encounter similar emotionally demanding situations after the rotation and when preceptors continue to use a shared language for observing and feedbacking caring behaviors. For example, students may be more likely to retain and transfer CARE-based behaviors if later rotations include explicit prompts to identify patients’ emotional concerns, respond to family conflict, explain uncertainty, and support patient participation in decisions. In contrast, gains may attenuate if subsequent clinical environments prioritize task completion over relational care, provide limited feedback on communication behaviors, or expose students to high workload and emotional stress without reflective support. This may be particularly relevant for dimensions such as patience, emotional regulation, and knowledge-based caring, which may require repeated real-case application before becoming stable professional habits.

Therefore, the present findings should be interpreted as evidence of immediate, self-reported caring-related improvement rather than proof of sustained professional formation. To support durability, curriculum designs should include longitudinal reinforcement across rotations, brief CARE-based reflection tools, repeated simulation or bedside micro-debriefing, and objective follow-up assessments.

### 4.5 Integration with established simulation frameworks

To position these findings within the broader simulation literature, it is useful to consider established frameworks such as the NLN Jeffries Simulation Framework, which emphasizes simulation design characteristics, educational practices, teacher and student factors, and outcomes. The Jeffries framework has been widely applied to teach clinical reasoning and technical skills, yet it does not explicitly specify how caring ability and caring behaviors should be embedded into unfolding scenarios or debriefing scripts. In this study, the CARE framework (Connect, Acknowledge, Restructure, Empower) was developed as a domain-specific scaffold that translates Watson’s and Swanson’s caring theories into observable, debriefable learning tasks across each simulation phase. Rather than replacing general simulation frameworks, CARE complements them by providing an explicit structure for caring-oriented simulation design in emotionally demanding specialties such as OBGYN. This perspective helps clarify how the present study relates to existing simulation frameworks: it applies a caring-specific scaffold within a general simulation design, offering a replicable approach for integrating humanistic care into unfolding case–based simulation.

## 5 Limitations

This study has several limitations.

First, this study adopted a prospective quasi-experimental design rather than a fully randomized controlled design. Although allocation was implemented at the individual level within rotation cohorts and baseline comparability was confirmed, students were still embedded in cohort-organized clinical rotations. Therefore, cohort-related clustering and shared teaching-context effects, such as common preceptors, clinical workload, case mix, teaching rhythm, and peer interaction, could not be fully excluded. The analysis treated participants as independent observations and did not model cohort effects using cluster-adjusted or multilevel methods. Future studies should consider individual randomization with stronger allocation concealment, cluster-randomized designs with an adequate number of clusters, or mixed-effects models to account for possible cohort-level dependence. In addition, the intervention was delivered as a bundled educational package; therefore, the independent effects of CARE-oriented content, unfolding simulation structure, pre-class preparation, and structured debriefing could not be isolated.

Second, the observed effect sizes for CAI and CBI were unusually large for a short-cycle educational intervention and should be interpreted cautiously. Such large effects may partly reflect immediate post-intervention measurement, novelty and Hawthorne effects, demand characteristics, students’ awareness of group assignment, and close conceptual overlap between the CARE-guided simulation content and the CAI/CBI constructs. Therefore, these findings should not be interpreted as definitive evidence that stable caring dispositions or real-world caring behaviors were transformed within a single 4-week rotation. Rather, they should be understood as short-term improvements in self-reported caring-related outcomes that require confirmation using more rigorous designs and objective outcome measures.

Third, outcome measures were primarily based on self-reported scales, which are subject to social desirability, response shift, and measurement reactivity. This concern is particularly relevant because participants in the intervention group were aware that they had received a novel caring-focused educational approach. As a result, improvements in CAI and CBI may partly reflect perceived learning gains or desirable responses rather than actual behavioral change in clinical practice. The absence of objective behavioral outcome measures, such as blinded OSCE ratings, standardized patient assessments, independent clinical observations, or patient/family evaluations, limits the ability to determine whether the observed improvements translated into real-world caring behaviors.

Fourth, this was a single-center convenience sample with a 4-week observation window. The study therefore captured immediate post-rotation outcomes only and could not determine whether caring-related gains persisted after students entered subsequent clinical rotations. Multicenter studies with delayed follow-up are needed to examine the durability, decay, and transfer of CARE-related learning across clinical contexts.

## Data Availability

The raw data supporting the conclusions of this article will be made available by the authors, without undue reservation.
